# Miscarriage, Preterm Delivery, and Stillbirth: Large Variations in Rates within a Cohort of Australian Women

**DOI:** 10.1371/journal.pone.0037109

**Published:** 2012-05-21

**Authors:** Alexis J. Hure, Jennifer R. Powers, Gita D. Mishra, Danielle L. Herbert, Julie E. Byles, Deborah Loxton

**Affiliations:** 1 Research Centre for Gender Health and Ageing, University of Newcastle, Callaghan, New South Wales, Australia; 2 School of Population Health, University of Queensland, Herston, Queensland, Australia; Université de Montréal, Canada

## Abstract

**Objectives:**

We aimed to use simple clinical questions to group women and provide their specific rates of miscarriage, preterm delivery, and stillbirth for reference. Further, our purpose was to describe who has experienced particularly low or high rates of each event.

**Methods:**

Data were collected as part of the Australian Longitudinal Study on Women's Health, a national prospective cohort. Reproductive histories were obtained from 5806 women aged 31–36 years in 2009, who had self-reported an outcome for one or more pregnancy. Age at first birth, number of live births, smoking status, fertility problems, use of *in vitro* fertilisation (IVF), education and physical activity were the variables that best separated women into groups for calculating the rates of miscarriage, preterm delivery, and stillbirth.

**Results:**

Women reported 10,247 live births, 2544 miscarriages, 1113 preterm deliveries, and 113 stillbirths. Miscarriage was correlated with stillbirth (*r* = 0.09, *P*<0.001). The calculable rate of miscarriage ranged from 11.3 to 86.5 miscarriages per 100 live births. Women who had high rates of miscarriage typically had fewer live births, were more likely to smoke and were more likely to have tried unsuccessfully to conceive for ≥12 months. The highest proportion of live preterm delivery (32.2%) occurred in women who had one live birth, had tried unsuccessfully to conceive for ≥12 months, had used IVF, and had 12 years education or equivalent. Women aged 14–19.99 years at their first birth and reported low physical activity had 38.9 stillbirths per 1000 live births, compared to the lowest rate at 5.5 per 1000 live births.

**Conclusion:**

Different groups of women experience vastly different rates of each adverse pregnancy event. We have used simple questions and established reference data that will stratify women into low- and high-rate groups, which may be useful in counselling those who have experienced miscarriage, preterm delivery, or stillbirth, plus women with fertility intent.

## Introduction

Reproduction involves a considerable risk of losing the offspring at some point during pregnancy or just after birth. Yet advances in reproductive medicine have facilitated a cultural expectation that every woman should be able to have a child [1]. Women who become pregnant also expect their pregnancy to go smoothly [1]. Hence, there appears to be a mismatch between the expectation and reality of human reproduction. Miscarriage (spontaneous abortion before 20 weeks gestation) is the most common of the adverse pregnancy outcomes. Though, there is no real agreement throughout the literature on just how frequently miscarriage occurs, with suggested rates of 8–20% [2], 31% [3], up to 50% [4], and higher [5]. Preterm delivery occurs in approximately 9.6% of all births globally [6] and is the leading cause of neonatal death [7]. _ENREF_3Stillbirths are rarer, but occur for about 3 million births during the third trimester of pregnancy each year [8]. In high income countries, 1 in every 200 women who reach 22 weeks gestation will have a stillborn baby [9].

From an evolutionary perspective, what we consider to be an adverse pregnancy outcome often represents biological screening [5] or natural selection. For example, more than half of all miscarried fetuses have genetic abnormalities [10], [11]. Many preterm deliveries occur because of intrauterine infection [12]. Up to one third of all stillborns will have detectable congenital and/or genetic abnormalities classified as the cause of death [13]. Within obstetrics there is a need to establish and reinforce the difference between ‘normal’ and ‘optimal’ (live, term birth) reproductive outcomes. Furthermore, what is ‘normal’ for one woman may not be the same as for another.

We aimed to summarise the reproductive histories of a cohort of Australian women into a clinical assessment tool. We provide reference data on the specific rates of miscarriage, preterm delivery, and stillbirth for different groups of Australian women, based on physical and lifestyle characteristics. We then describe who has experienced low or high rates of each adverse event. In doing so, women who experience miscarriage, preterm delivery, or stillbirth may then be counselled according to data from women more similar to themselves.

**Table 1:** Clinical questions and responses for summarising women's reproductive histories.

Clinical Questions	Response categories	Relevant to rate of:
Q1.How many live births have you had?	□0□1□2□3□≥4	MiscarriagePreterm delivery
Q2.How old were you when you had your first birth?	□No births□14–19.99□20–24.99□25–29.99□30–36	Stillbirth
Q3.Have you ever tried unsuccessfully for 12 months or more to get pregnant?	□No□Yes	MiscarriagePreterm delivery
Q4.Have you ever used IVF?	□No□Yes	Preterm delivery
Q5.Which of the following best describes your current smoking status?	□Never smoked□Quit smoking□<daily smoking□Daily smoking	Miscarriage
Q6.Which of the following is your highest qualification?	□Didn't complete year 12□Year 12 or equivalent (includes apprenticeship, trade, certificate, or diploma)□University degree	Preterm delivery
Q7.Which of the following best describes your current level of physical activity?	□Sedentary□Low□Moderate□High	Stillbirth

**Table 2:** Physical, lifestyle and reproductive characteristics for a national cohort of Australian women.

	All	Number of Live Births				
		0	1	2	3	≥4
	*N* = 5806	610	1629	2421	885	261
Age (yr) (mean ±SD)	33.8±1.5	33.5±1.4	33.6±1.4	33.9±1.4	34.1±1.4	34.3±1.5
Age at first birth^a^ (yr) (mean ±SD) (n = 5191)	27.7±4.1	b	30.3±3.6	27.5±3.4	24.9±3.5	22.4±3.4
Area of residence at baseline (survey 1, 1996) (n, %)						
Urban	3110 (53.6%)	390 (63.9%)	936 (57.5%)	1286 (53.1%)	404 (45.7%)	94 (36.0%)
Rural	2469 (42.5%)	203 (33.3%)	641 (39.4%)	1048 (43.3%)	428 (48.4%)	149 (57.1%)
Remote	227 (3.9%)	17 (2.8%)	52 (3.2%)	87 (3.6%)	53 (6.0%)	18 (6.9%)
Weight (kg) (mean ±SD) (n = 5804)	71.3±16.7	71.0±17.0	70.6±17.4	71.3±16.0	71.7±16.5	75.3±18.4
Height (cm) (mean ±SD) (n = 5742)	166.1±7.1	166.1±6.9	166.0±7.1	166.2±7.0	165.8±7.3	166.0±6.9
Body Mass Index (BMI in kg/m^2^) (mean ±SD) (n = 5741)	25.9±5.8	25.7±5.7	25.6±6.0	25.8±5.6	26.0±5.7	27.3±6.6
BMI category (n, %)						
Underweight (<18.50)	161 (2.8%)	12 (2.0%)	59 (3.7%)	64 (2.7%)	21 (2.4%)	5 (2.0%)
Normal weight (18.50–24.99)	2956 (51.5%)	332 (54.8%)	852 (52.8%)	1217 (50.8%)	441 (50.6%)	114 (44.9%)
Overweight (25.00–29.99)	1506 (26.2%)	156 (25.7%)	399 (24.7%)	658 (27.5%)	233 (26.7%)	60 (23.6%)
Obese (≥30.00)	1118 (19.5%)	106 (17.5%)	305 (18.9%)	455 (19.0%)	177 (20.3%)	75 (29.5%)
Marital status (n, %) (n = 5802)						
Married or de facto	5080 (87.6%)	388 (63.7%)	1402 (86.1%)	2253 (93.1%)	804 (91.0%)	233 (89.3%)
Separated, divorced, or widowed	352 (6.1%)	33 (5.4%)	108 (6.6%)	124 (5.1%)	63 (7.1%)	24 (9.2%)
Never married	370 (6.4%)	188 (30.9%)	118 (7.3%)	43 (1.8%)	17 (1.9%)	4 (1.5%)
Education, highest qualification (n, %)						
<Yr 12 (Higher School Certificate) or equivalent	510 (8.8%)	37 (6.1%)	110 (6.8%)	188 (7.8%)	117 (13.2%)	58 (22.2%)
Yr 12, apprenticeship, trade, certificate or diploma	2504 (43.1%)	223 (36.6%)	633 (38.9%)	1089 (45.0%)	430 (48.6%)	129 (49.4%)
Undergraduate or higher university degree	2792 (48.1%)	350 (57.4%)	886 (54.4%)	1144 (47.3%)	338 (38.2%)	74 (28.4%)
Smoking status (n, %) (n = 5804)						
Never smoked	3609(62.2%)	299 (49.1%)	1048 (64.3%)	1568 (64.8%)	553 (62.5%)	141 (54.0%)
Quit smoking	1332(23.0%)	164 (26.9%)	377 (23.1%)	527 (21.8%)	192 (21.7%)	72 (27.6%)
Smoking<daily	195 (3.4%)	44 (7.2%)	37 (2.3%)	78 (3.2%)	27 (3.1%)	9 (3.5%)
Daily smoking	668 (11.5%)	102 (16.8%)	167 (10.3%)	247 (10.2%)	113 (12.8%)	39 (14.9%)
Alcohol intake (n, %) (n = 5801)						
Abstinence	762 (13.1%)	62 (10.2%)	244 (15.0%)	281(11.6%)	125 (14.1%)	50 (19.2%)
Rarely or low risk (1–2 drinks^c^ per day)	4792 (82.6%)	497 (81.5%)	1330 (81.8%)	2034 (84.1%)	727 (82.2%)	204 (78.2%)
Risky or high risk (≥3 drinks per day)	247 (4.3%)	51 (8.4%)	53 (3.3%)	103 (4.3%)	33 (3.7%)	7 (2.7%)
History of risky or high risk drinking^c^	2275 (39.2%)	322 (52.8%)	702 (43.1%)	853 (35.2%)	305 (34.5%)	93 (35.6%)
History of emotional or physical abuse (n = 5729)	3367 (58.8%)	281 (46.7%)	949 (59.3%)	1498 (62.5%)	509 (58.2%)	130 (50.6%)
Physical activity (n, %) (n = 5755)						
Sedentary	911 (15.8%)	78 (12.9%)	247 (15.3%)	377 (15.7%)	159 (18.2%)	50 (19.3%)
Low	2447 (42.5%)	212 (34.9%)	715 (44.2%)	1047 (43.7%)	363 (41.5%)	110 (42.5%)
Moderate	1175 (20.4%)	135 (22.2%)	335 (20.7%)	488 (20.4%)	168 (19.2%)	49 (18.9%)
High	1222 (21.2%)	182 (30.0%)	322 (19.9%)	484 (20.2%)	184 (21.1%)	50 (19.3%)
Fertility problems, tried unsuccessfully for ≥12 mo (n, %) (n = 5804)	1065 (18.4%)	120 (19.7%)	385 (23.6%)	397 (16.4%)	128 (14.5%)	35 (13.4%)
Have used In Vitro Fertilisation (n, %) (n = 5804)	269 (4.6%)	39 (6.4%)	130 (8.0%)	72 (3.0%)	25 (2.8%)	3 (1.2%)
Have had a multiple birth^d^ (n, %)	149 (2.6%)	0	23 (1.4%)	50 (2.1%)	48 (5.4%)	28 (10.7%)
Have had ≥1 adverse pregnancy event(s) (n, %)						
Miscarriage	1757 (30.3%)	226 (37.0%)	399 (24.5%)	684 (28.3%)	332 (37.5%)	116 (44.4%)
Preterm delivery (n = 5791)	826 (14.3%)	0	226 (13.9%)	353 (14.6%)	179 (20.2%)	68 (26.3%)
Stillbirth	95 (1.6%)	4 (0.7%)	22 (1.4%)	35 (1.4%)	20 (2.3%)	14 (5.4%)
None of the above (n = 5791)	3468 (59.9%)	381 (62.6%)	1067 (65.6%)	1480 (61.4%)	437(49.4%)	103 (39.8%)

a Live birth or stillbirth. Minimum and maximum age at first birth: 14 to 36 years.

b n = 606 have not had a live birth. Age for n = 2 stillbirths: 25.5±10.6 years. n = 2 missing age at stillbirth.

c 1 standard drink = 10 g alcohol. Risky drinking defined as ≥15 standard drinks per week (equivalent to 2.5 bottles of wine or 14.5 cans of beer). High-risk drinking defined as ≥28 drinks per week. Definitions from National Health and Medical Research Council (2001), *Australian Alcohol Guidelines: Health Risks and Benefits*.

d Includes n = 5 sets of triplets and 1 woman had 2 sets of twins.

**Table 3:** Spearman's rank correlation coefficients for pregnancy outcomes in Australian women.

		Miscarriage		Preterm delivery		Stillbirth	
	*n*	*r*	*P*	*r*	*P*	*r*	*P*
**Preterm delivery**	5791	0.04	0.007				
**Stillbirth**	5806	0.09	<0.001	0.12	<0.001		
**Live birth**	5806	0.06	<0.001	0.15	<0.001	0.05	<0.001

**Table 4:** Reference rates of miscarriage for different groups of Australian women.

Clinical Questions & Responses			Miscarriage rate per 100 live births (95% CI)		Rate Ranking^a^	% who had ≥1 miscarriage	n
Number of live births	Smoking status	Fertility problems					
0	Never	No	n = 73	*Rates not calculable for 0 live births. Total number of miscarriages presented.*	High	26.8	231
0	Never	Yes	n = 79		High	77.9	68
0	Quit	No	n = 56		High	28.8	139
0	Quit	Yes	n = 32		High	80.0	25
0	<daily	No	n = 7		High	16.2	37
0	Daily	No	n = 34		High	29.6	81
0	Daily	Yes	n = 26		High	75.0	20
1	Never	No	23.7	(20.8, 26.8)	Moderate	18.7	793
1	Never	Yes	59.6	(53.3, 65.7)	High	36.9	255
1	Quit	No	29.6	(24.4, 35.2)	Moderate	23.7	291
1	Quit	Yes	65.1	(54.1, 75.1)	High	32.6	86
1	<daily	No	23.3	(9.9, 42.3)	Moderate	23.3	30
1	Daily	No	35.4	(27.2, 44.2)	Moderate	26.2	130
1	Daily	Yes	86.5	(71.2, 95.5)	High	45.9	37
2	Never	No	15.5	(14.1, 16.9)	Low	24.2	1310
2	Never	Yes	35.8	(31.6, 40.1)	Moderate	44.0	257
2	Quit	No	16.2	(13.8, 18.9)	Low	23.9	431
2	Quit	Yes	50.0	(42.7, 57.3)	High	56.3	96
2	<daily	No	12.3	(7.3, 19.0)	Low	17.4	69
2	Daily	No	23.1	(19.2, 27.4)	Moderate	30.2	212
2	Daily	Yes	41.4	(30.0, 53.8)	High	42.9	35
3	Never	No	15.3	(13.5, 17.3)	Low	34.7	473
3	Never	Yes	25.0	(19.7, 31.0)	Moderate	47.5	80
3	Quit	No	17.4	(14.1, 21.0)	Low	33.7	163
3	Quit	Yes	36.8	(26.7, 47.8)	Moderate	58.6	29
3	<daily	No	25.0	(15.5, 36.6)	Moderate	54.2	24
3	Daily	No	18.9	(14.6, 23.9)	Low	39.2	97
3	Daily	Yes	22.9	(12.0, 37.3)	Moderate	43.8	16
≥4	Never	No	16.3	(13.3, 19.8)	Low	49.2	122
≥4	Never	Yes	11.3	(5.3, 20.3)	Low	31.6	19
≥4	Quit	No	20.6	(15.9, 26.0)	Moderate	47.5	61
≥4	Quit	Yes	26.5	(14.9, 41.1)	Moderate	63.6	11
≥4	Daily	No	16.7	(11.2, 23.5)	Low	25.7	35

a Low<20, Moderate = 20–40, High>40 miscarriages per 100 live births.

**Table 5:** Reference rates of preterm delivery for different groups of Australian women.

Clinical Questions				% of live births born Preterm (95% CI)		Rate Ranking^a^	% who had ≥1 preterm delivery	n
Number of live births	Fertility problems	IVF	Level of education					
1	No	No	<yr 12 or equivalent	21.0	(12.7, 31.5)	High	19.8	81
1	No	No	Yr 12 or equivalent	14.0	(10.9, 17.5)	High	12.8	453
1	No	No	University degree	11.3	(9.1, 13.9)	Moderate	11.0	707
1	Yes	No	<yr 12 or equivalent	24.0	(9.4, 45.1)	High	20.0	25
1	Yes	No	Yr 12 or equivalent	17.2	(10.9, 25.4)	High	17.2	116
1	Yes	No	University degree	16.2	(10.1, 24.2)	High	16.2	117
1	Yes	Yes	Yr 12 or equivalent	32.3	(20.9, 45.3)	High	27.4	62
1	Yes	Yes	University degree	26.2	(15.8, 39.1)	High	19.7	61
2	No	No	<yr 12 or equivalent	11.9	(8.6, 16.0)	Moderate	19.8	162
2	No	No	Yr 12 or equivalent	9.2	(7.9, 10.6)	Moderate	13.6	903
2	No	No	University degree	7.8	(6.6, 9.1)	Low	12.0	953
2	Yes	No	<yr 12 or equivalent	18.8	(8.9, 32.6)	High	29.2	24
2	Yes	No	Yr 12 or equivalent	13.5	(9.9, 17.8)	Moderate	20.3	153
2	Yes	No	University degree	10.8	(7.5, 14.8)	Moderate	17.0	153
2	Yes	Yes	Yr 12 or equivalent	27.6	(16.7, 40.9)	High	36.7	30
2	Yes	Yes	University degree	21.4	(12.5, 32.9)	High	25.7	35
3	No	No	<yr 12 or equivalent	12.0	(8.5, 16.2)	Moderate	22.0	100
3	No	No	Yr 12 or equivalent	9.4	(7.7, 11.3)	Moderate	19.0	358
3	No	No	University degree	8.0	(6.2, 9.9)	Low	14.5	297
3	Yes	No	<yr 12 or equivalent	22.2	(10.1, 39.2)	High	41.7	12
3	Yes	No	Yr 12 or equivalent	15.3	(10.3, 21.4)	High	32.2	59
3	Yes	No	University degree	22.5	(14.9, 31.9)	High	35.3	34
3	Yes	Yes	Yr 12 or equivalent	30.6	(16.3, 48.1)	High	41.7	12
≥4	No	No	<yr 12 or equivalent	13.7	(9.5, 18.8)	Moderate	33.3	51
≥4	No	No	Yr 12 or equivalent	10.4	(7.8, 13.5)	Moderate	25.2	111
≥4	No	No	University degree	7.8	(4.8, 11.7)	Low	20.6	63
≥4	Yes	No	Yr 12 or equivalent	13.2	(6.2, 23.6)	Moderate	37.5	16
≥4	Yes	No	University degree	4.9	(0.6, 16.5)	Low	10.0	10

a Low<9, Moderate = 9–14, High>14 preterm deliveries per 100 live births.

**Table 6:** Reference rates of stillbirth for different groups of Australian women.

Clinical Questions		Stillbirth rate per 1000 live births (95% CI)		Rate Ranking^a^	% who had ≥1 stillbirth	n
Age at first birth	Physical activity					
14–19.99	Sedentary	30.6	(11.3, 65.4)	High	5.6	72
14–19.99	Low	38.9	(20.9, 65.6)	High	8.4	119
14–19.99	Moderate	6.5	(0.2, 35.6)	Low	1.9	53
14–19.99	High	6.0	(0.2, 32.9)	Low	1.8	56
20–24.99	Sedentary	22.9	(11.1, 41.8)	High	3.4	176
20–24.99	Low	14.7	(8.1, 24.5)	High	3.2	373
20–24.99	Moderate	7.0	(1.4, 20.2)	Low	1.8	169
20–24.99	High	7.6	(2.1, 19.5)	Low	1.8	223
25–29.99	Sedentary	12.8	(5.9, 24.1)	Moderate	2.4	339
25–29.99	Low	7.9	(4.4, 13.0)	Low	1.5	904
25–29.99	Moderate	5.5	(1.8, 12.9)	Low	1.2	434
25–29.99	High	6.2	(2.3, 13.3)	Low	1.1	476
30–36	Sedentary	5.7	(0.6, 20.4)	Low	0.8	245
30–36	Low	8.5	(4.1, 15.5)	Moderate	1.0	838
30–36	Moderate	9.3	(3.0, 21.6)	Moderate	1.0	383
30–36	High	10.2	(2.8, 25.9)	Moderate	1.4	284

a Low<8, Moderate = 8–13, High>13 stillbirths per 1000 live births.

## Materials and Methods

Data were collected as part of the Australian Longitudinal Study on Women's Health (ALSWH). Full details of the prospective study design and recruitment have been reported elsewhere [14]–[16]. Briefly, the ALSWH recruited 14,247 women aged 18 to 23 years at the baseline survey in 1996. Potential participants were randomly selected from the national Medicare database, except that women from non-urban areas were intentionally over-sampled [14]. An invitation to participate was mailed out and those who consented were deemed broadly representative of women of the same age within the Australian population [16]. _ENREF_9Ethics approvals were obtained from the Human Research Ethics Committees of the Universities of Newcastle and Queensland, and written informed consent was provided by participants.

This paper presents self-reported reproductive history data collected at Survey 5 in 2009, when the participants were 31 to 36 years. Data from earlier surveys were accessed as needed, for example, to describe area of residence at baseline. Survey 5 responses were received from 8200 women in 2009; 58% of those who completed the baseline survey in 1996. Compared to non-responders, more women who completed Survey 5 had never smoked (54% vs. 45%) and had ≥12 years education (70% vs. 65%) at baseline. However, women who completed Survey 5 were not meaningfully different to non-responders in terms of age, marital status, or area of residence at baseline.

Participants were excluded from these analyses if: (i) they had never been pregnant (n = 2098), (ii) they were currently pregnant but reported no previous pregnancies (n = 182), (iii) their pregnancy data were reported inconsistently at two or more questions (n = 53), (iv) they were unsure if they were currently pregnant and reported no previous pregnancy (n = 35), or (v) their pregnancy outcomes (miscarriage, live birth, and stillbirth) were all missing (n = 26). Women who had complete data for number of miscarriages, stillbirths and live births but were missing the total number of preterm deliveries (n = 15) were included in the analyses. Women who reported having had a termination were included in the analysis, but termination data have been reported elsewhere [17]. Therefore, data for 5806 women who reported an outcome for at least one pregnancy have been included. Last observation carried forward was used when the participant had answered the pregnancy outcome questions at Survey 4 in 2006, but not at Survey 5 in 2009; 0.8% of the pregnancy outcome data (not cases) were replaced.

Participants were asked ‘How many times have you had each of the following?’ with live birth, stillbirth and miscarriage listed thereafter. The response categories were ordinal, up to ‘5 or more’. Where ‘5 or more’ live births were reported (n = 6), the dates of birth for the children were cross-referenced to determine the total number of live births, after subtracting any stillbirths. Thirty one women reported ‘5 or more’ miscarriages and these were counted as five in the analyses. Women were asked if they had ever experienced a ‘premature birth’ and for which child this occurred. No definition of premature birth was given at Survey 5 in 2009, however, Survey 4 in 2006 had specified ‘36 weeks or less’.

Descriptive statistics were used to summarise maternal physical, lifestyle and reproductive characteristics for all women, and were categorised according to number of live births. The rate of miscarriage was calculated per 100 live births; stillbirths per 1000 live births. The rate of preterm delivery was calculated as a proportion of live births. Live births were chosen to reflect an ‘optimal’ pregnancy outcome. Correlations between pregnancy outcomes were assessed using Spearman's rank tests with Bonferroni correction for multiple comparisons.

Decision analysis was undertaken to separate women into groups, prior to calculating the specific rates of miscarriage, preterm delivery, and stillbirth. Multivariate backward stepwise regressions (Poisson, *P*<0.05) were used to select the clinical questions that would best stratify the women into groups. Number of live births, maternal age at first birth, body mass index (BMI) [18], marital status, level of education, smoking status [19], alcohol intake (including history of risky drinking) [20], history of emotional or physical abuse, physical activity level, fertility problems (having tried unsuccessfully to conceive for ≥12 months, at any age) [21], and use of *in vitro* fertilisation (IVF) were all considered. Table 1 summarises the clinical questions and response categories that best distinguished groups of women for each outcome. The rates of miscarriage, preterm delivery, and stillbirth were then calculated for each group of women, relative to the number of live births. The rates of each adverse outcome were assigned a ranking of low, moderate or high, depending on how they compared to the previously published rates of miscarriage [2], preterm birth [22], and stillbirth [22]. Only groups with n≥10 women are reported in the results. Analyses were performed using Intercooled Stata, version 11 (StataCorp, USA).

## Results

In total the 5806 women reported 2544 miscarriages, 113 stillbirths and 10,247 live births. Preterm deliveries were reported for 10.7% (n = 1113) of all births (live- and stillbirths). The overall rate of miscarriage was 25 per 100 live births and the stillbirth rate was 11 per 1000 live births.

Approximately 70% of women recorded one or two live births by a mean age of 33.8±1.4 years (Table 2). Two in every five women (n = 2540) had experienced at least one miscarriage, preterm delivery, and/or stillbirth. Of these 35% (n = 894) had experienced two or more of these events. Statistically significant correlations were observed for miscarriages, preterm deliveries, stillbirths and live births (Table 3). The strongest correlations were for preterm delivery, which must also result in either a live- or stillbirth.

Table 4 is presented in the same order as the clinical questions and responses categories in Table 1, rather than by miscarriage rate. The group who had one live birth, smoked daily and had tried unsuccessfully to conceive for ≥12 months had the highest calculable miscarriage rate at 86.5 miscarriages per 100 live births. There was an 8-fold difference in the group with the lowest versus the highest calculable rate for miscarriage. Eighty per cent of women who had no live births, had quit smoking or had never smoked, and had tried unsuccessfully to conceive for ≥12 months had experienced one or more miscarriage(s). The most consistent differences in the groups who had low compared to high rates of miscarriage were having had a greater number of live births and an absence of fertility problems. Women in the low-rate miscarriage groups were also less likely to have smoked compared to the high-rate groups.

There was a seven-fold difference in the group with the lowest versus the highest proportion of live preterm deliveries (Table 5). The highest rate of preterm deliveries (32.2%) occurred in the group of women who had one live birth, had tried unsuccessfully to conceive for ≥12 months, had used IVF and had an education level at or equivalent to having finished Year 12. The most consistent differences in the groups who had low compared to high rates of preterm delivery were not having fertility problems or having used IVF treatment. The number of live births and level of education were also higher in women with low rates of preterm delivery.

The group of women who were 25–29.99 years at their first birth and who reported moderate physical activity had the lowest rate of stillbirths, at 5.5 per 1000 live births (Table 6). Women who were 14–19.99 years at their first birth and reported low physical activity had a rate that was seven-fold higher, at 38.9 stillbirths per 1000 live births. Physical activity level was higher in women with low rates of stillbirth. Both low and high rates of stillbirth were observed for the youngest age at first birth category (14–19.99 years).

## Discussion

This paper has taken a novel approach to analysing the reproductive histories of Australian women, focusing on the rates of miscarriage, preterm delivery, and stillbirth. We have demonstrated that different groups of women experience vastly different rates of each event and have established a series of clinical questions (Table 1) that will stratify women into low- and high-rate groups for comparison. Women should be counselled appropriately when they experience an adverse pregnancy event, and this may now include putting their reproductive outcomes in the context of what other women who are similar to them have experienced. Furthermore, women with fertility intentions who fall within high-risk categories prior to conception may also be counselled on lifestyle changes as a primary prevention measure. Rather than using this dataset to test what the predictors of adverse pregnancy outcomes may be (there are many papers that already do this [23]–[25]) we have devised a method to summarise the actual number of events that different groups of Australian women have experienced over 20 years. In addition, we have shown that there is a small but significant correlation between these adverse pregnancy outcomes. In particular, women who have experienced miscarriage are also more likely to have had a stillbirth.

Age at first birth, number of live births, smoking status, fertility problems, use of IVF, and level of education and physical activity were the variables that best separated women into low or very high rates of miscarriage, preterm delivery, and stillbirth. Many other variables that have been shown throughout the literature to be associated with adverse birth outcomes were tested for inclusion, but were less, if at all, significant. An absence of fertility problems was the most striking variable for low rates of miscarriage and preterm delivery, which is in line with other published work_ENREF_17 [26], [27]. A greater number of live births was also strongly associated with better reproductive outcomes. Miranda *et al.* (2011) recently showed that higher rates of adverse outcomes in nulliparous women are partly attributed to higher-risk women not having a subsequent live birth, either because of poor fertility or by choice [28].

The national rate of recognised miscarriage from this survey was 1 for every 4 live births, a figure not previously available within Australia because data on miscarriage is not recorded and published systematically from state-based health datasets. However, there was huge variation in the calculable rates, which would be higher again if the groups with no live births were included. This variation in the rate of miscarriage by group may help to explain, in part, why there are so many different rates of miscarriage cited throughout the literature [2]–[5]. ALSWH participants were only able to report miscarriages for pregnancies that they had recognised. Wilcox *et al.* (1988) have previously demonstrated that 22% of pregnancies end before they are clinically detected [3]. Therefore, the true national rate of miscarriage is likely to be higher than 1 miscarriage for every 4 live births.

The rate of preterm delivery was remarkably similar for our data compared to global estimates [6] (9.7 vs. 9.6% respectively), but was slightly higher than the most recent national statistic reported as 8.2% of all births in 2009 [22]. This may be because no definition was provided in Survey 5 of the ALSWH, resulting in a misunderstanding of what was meant by ‘premature birth’. However, there is also some evidence to suggest that the rate of preterm delivery, from both spontaneous rupture of membranes and medical intervention, has increased [7]. The rate of self-reported stillbirth was also higher in our dataset than national figures (11.0 vs. 7.8 [22] per 1000 live births). In Australia, a stillbirth is defined as a fetal death (including terminations), weighing at least 400 grams at delivery or whose period of gestation was at least 20 weeks [22]. The Australian Government has paid a maternity allowance for all live- and stillbirths since February 1996 [29], whereas miscarriages do not result in any Government payment. This is one way to differentiate a late miscarriage from a stillbirth.

Our study relies on the participants having accurately recorded their obstetric events in much the same way as clinicians rely on patients to provide an accurate description of their medical history. Reproductive histories from medical records can only serve as another imperfect source of information and not as a gold standard [30]. The reliability of self -reported reproductive histories has been assessed against medical records in one study of 754 women [30]. Olsen *et al.* (1997) showed very high reliability measures and correlations between the two data sources (self-report and medical records) for number of live births (*k* = 1.0), number of previous pregnancies (*k* = 0.9), gestational age at birth (*r* = 0.8) and number of miscarriages (*k* = 0.7).

Having data on the reproductive outcomes for women only up to the age of 36 years is both a strength and limitation of this study. The rates presented here reflect reproductive outcomes before the ‘high-risk’ years [31]. Therefore, these reference data should only be applied to women who are 36 years or younger. The 58% response rate at Survey 5 of the ALSWH (2009), compared to baseline (1996), may have introduced bias into our results. Women who smoke are more likely to experience miscarriage [32], preterm delivery [33], and stillbirth [24]. We had 9% fewer women who had ever smoked participate at Survey 5, thus, the true rates of each event may be higher than we have calculated. A further limitation is that any paternal contribution to pregnancy outcome cannot be accounted for in this study.

The experience of miscarriage, preterm delivery, and stillbirth is generally regarded as very traumatic for those involved. Forty per cent of the women in this cohort who had been pregnant had experienced one or more miscarriage, preterm delivery, and/or stillbirth, by about 34 years of age. The reproductive histories of young Australian women demonstrate that reproduction is inherently risky, but more so for some than for others. Miscarriage, preterm delivery and stillbirth are programmed into our physiology and often represent biological screening when something is amiss. We have summarised Australian data into a clinical tool that service providers can use to evaluate a woman's reproductive history or to counsel them before or after an adverse pregnancy event has occurred. When risks are high, efforts should be made to minimise the chances of an adverse pregnancy outcome by providing evidence-based lifestyle interventions, such as smoking cessation.
